# Beyond Chronic Rhinosinusitis: Multisystem Granulomatosis With Polyangiitis Presenting With Cavitary Pulmonary Nodules and Sinonasal Destruction

**DOI:** 10.7759/cureus.114445

**Published:** 2026-08-12

**Authors:** Kian Memari, Emily Reinoso, Josue P Boutros, Maria J Morales, Anthony Nazur, Daniela Delphus, Lissette P Lazo, Shane Williams, Peter Cohen

**Affiliations:** 1 Family Medicine, Palmetto General Hospital, Miami, USA; 2 General Medicine, Nova Southeastern University Dr. Kiran C. Patel College of Osteopathic Medicine, Fort Lauderdale, USA; 3 General Medicine, Ross University School of Medicine, Miami, USA; 4 General Medicine, Nova Southeastern University Dr. Kiran C. Patel College of Osteopathic Medicine, Clearwater, USA; 5 Family Medicine, Nova Southeastern University Dr. Kiran C. Patel College of Osteopathic Medicine, Fort Lauderdale, USA

**Keywords:** c-anca, cavitary pulmonary nodules, chronic rhinosinusitis, granulomatosis with polyangiitis, sinonasal destruction, small-vessel vasculitis, stroke mimics

## Abstract

Granulomatosis with polyangiitis (GPA) is a rare necrotizing vasculitis of small- to medium-sized vessels characterized by granulomatous inflammation, most commonly involving the upper respiratory tract, lungs, and kidneys. Early manifestations are frequently non-specific and may resemble chronic allergic or infectious rhinosinusitis, delaying recognition until destructive sinonasal, pulmonary, renal, neurologic, or cutaneous involvement emerges. Cutaneous vasculitis and cavitary pulmonary nodules further complicate the diagnostic process by raising concern for infection or malignancy.

We report a 69-year-old woman with years of refractory nasal congestion and recurrent epistaxis who developed progressive sinonasal obstruction, septal perforation, palpable purpura, peripheral sensory symptoms, renal urinary abnormalities, and bilateral cavitary pulmonary nodules. Laboratory evaluation demonstrated PR3-ANCA/c-ANCA positivity, elevated inflammatory markers, anemia of inflammation, mild renal dysfunction, and urinalysis with proteinuria and microscopic hematuria. Computed tomography demonstrated destructive sinonasal disease and bilateral cavitary pulmonary nodules. Nasal biopsy showed necrotizing granulomatous inflammation with small to medium vessel vasculitis, confirming GPA. High-dose systemic glucocorticoids and rituximab-based induction therapy were initiated with *Pneumocystis jirovecii* pneumonia prophylaxis and multidisciplinary rheumatology, otolaryngology, pulmonology, and nephrology follow-up.

This case is not presented as a unique manifestation of GPA but as an educational reminder that chronic rhinosinusitis becomes a diagnostic trap when accompanied by epistaxis, septal destruction, pulmonary cavitation, purpura, neuropathic symptoms, or urinary abnormalities. Earlier recognition of this pattern may prevent irreversible organ damage.

## Introduction

Chronic nasal congestion and recurrent epistaxis are among the most common complaints encountered in outpatient and otolaryngologic practice and are frequently attributed to allergic rhinitis, chronic sinusitis, or nasal polyposis. In rare cases, however, these symptoms may represent the earliest manifestations of systemic vasculitis [1].

Granulomatosis with polyangiitis (GPA), formerly known as Wegener’s granulomatosis, is a rare autoimmune disease characterized by necrotizing granulomatous inflammation and vasculitis affecting small- to medium-sized blood vessels. A worldwide meta-analysis estimated the pooled incidence of antineutrophil cytoplasmic antibody (ANCA)-associated vasculitis at 17.2 per million person-years and pooled prevalence at 198.0 per million persons, underscoring the rarity of these disorders in general clinical practice [1].

GPA belongs to the spectrum of ANCA-associated vasculitides, along with microscopic polyangiitis and eosinophilic GPA [2]. GPA most frequently involves the upper respiratory tract, lungs, and kidneys but may also affect the skin, peripheral nerves, joints, and eyes [3]. Delayed diagnosis can result in destructive sinonasal disease, pulmonary cavitation, renal failure, and increased mortality. Pulmonary nodules, including cavitary nodules, are characteristic but diagnostically challenging because they can mimic infection, malignancy, sarcoidosis, and other inflammatory diseases [3].

The disease is most commonly associated with antibodies directed against proteinase 3 (PR3-ANCA or c-ANCA), which promote neutrophil activation, endothelial injury, granuloma formation, and tissue destruction [4]. Because early symptoms often mimic benign inflammatory conditions, GPA is frequently underrecognized in its initial stages.

The educational gap addressed by this case is delayed recognition of GPA when long-standing sinonasal complaints anchor the diagnostic process toward benign rhinosinusitis. We present a patient with chronic refractory sinonasal disease, septal perforation, pulmonary cavitation, cutaneous vasculitis, neuropathic symptoms, and urinary abnormalities, emphasizing the clinical triggers that should prompt earlier multisystem evaluation.

## Case presentation

A 69-year-old woman presented with persistent nasal congestion, recurrent epistaxis, throat discomfort, and pruritic, scaly lesions on both hands. Her symptoms had progressed over several years and were refractory to antihistamines and intranasal corticosteroids. She denied fever, weight loss, night sweats, dysphagia, or hemoptysis.

Her medical history was notable for chronic rhinitis, recurrent epistaxis, atopic dermatitis of the hands, and a remote spontaneous pneumothorax. Surgical history included prior nasal surgery with biopsy, partial hysterectomy, appendectomy, and tympanostomy tube placement. She had a remote smoking history and quit 25 years earlier.

**Figure 1:**
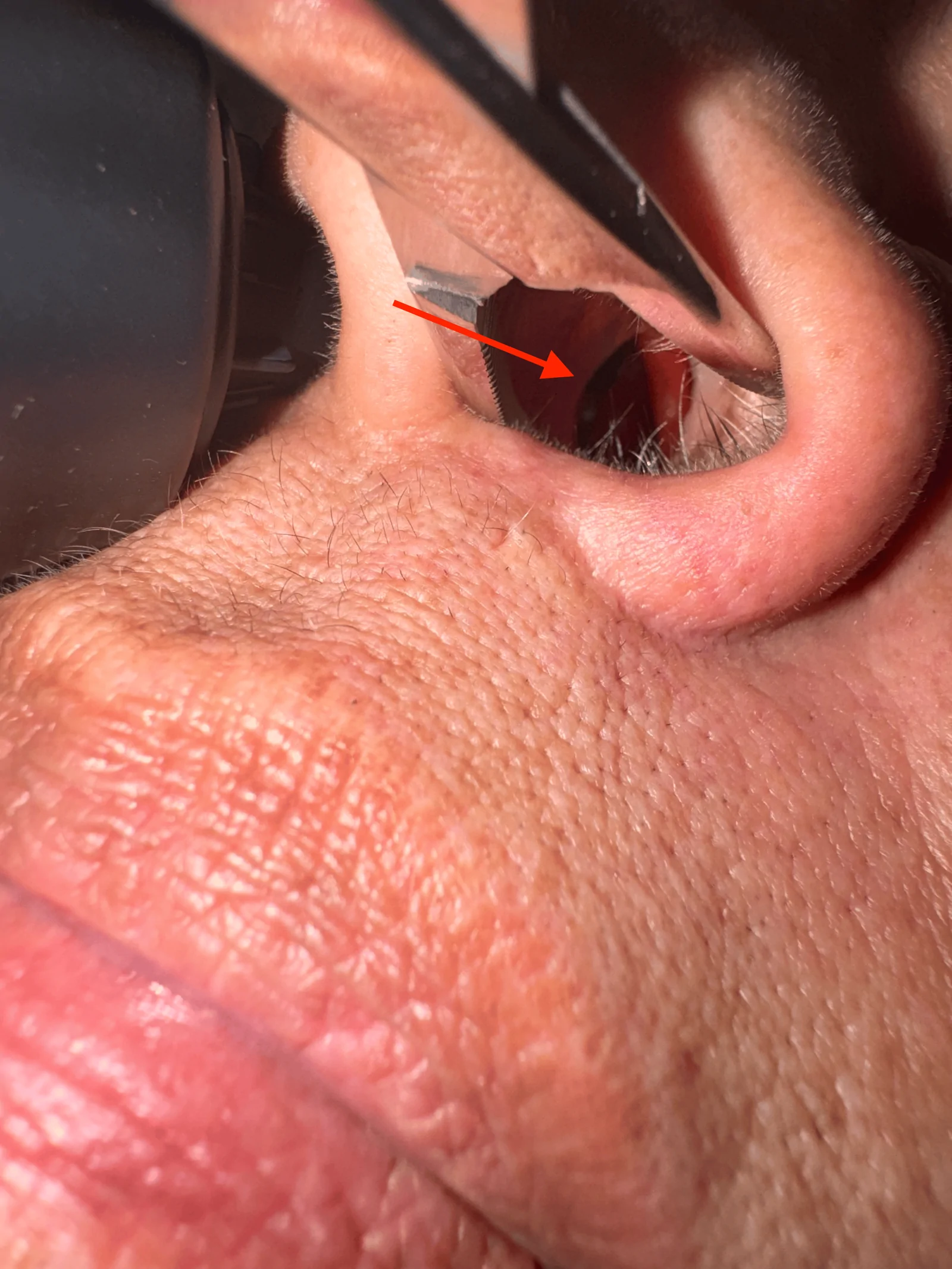
Nasal septal perforation Otolaryngologic examination demonstrating edematous nasal mucosa with nasal septal perforation (red arrow).

On examination, vital signs were stable. Otolaryngologic examination revealed edematous nasal mucosa with septal perforation (Figure 1). Dermatologic examination demonstrated palpable purpura on the lower extremities, consistent with small vessel vasculitis; however, clinical photographs were not obtained. Neurologic examination revealed decreased distal sensation in the lower extremities without focal deficits. The remainder of the examination was unremarkable.

A structured clinical timeline was added to improve reproducibility (Table 1). 

**Table 1:** Clinical timeline from symptom onset to diagnosis and early treatment response ANCA, antineutrophil cytoplasmic antibody; CRP, C-reactive protein; CT, computed tomography; ESR, erythrocyte sedimentation rate.

Time point	Clinical event
Several years before diagnosis	Persistent nasal congestion and recurrent epistaxis began; symptoms were treated as chronic rhinitis/rhinosinusitis.
Months before diagnosis	Progressive nasal obstruction and recurrent epistaxis persisted despite antihistamines and intranasal corticosteroids; pruritic hand lesions and lower-extremity purpura were noted.
Index presentation	Examination revealed edematous nasal mucosa, septal perforation, palpable purpura, and distal sensory loss.
Initial diagnostic workup	Laboratory testing showed PR3-ANCA/c-ANCA positivity, elevated ESR/CRP, mild anemia, a creatinine of 1.4 mg/dL, and proteinuria and microscopic hematuria on urinalysis.
Imaging workup	Chest radiography and CT showed bilateral nodular/cavitary pulmonary disease; sinus CT showed mucosal thickening, soft tissue disease, and destructive nasal septal changes.
Diagnostic confirmation	Nasal biopsy demonstrated necrotizing granulomatous inflammation and vasculitis involving small- and medium-sized vessels.
Treatment initiation	High-dose systemic corticosteroids and rituximab-based induction therapy were initiated with trimethoprim-sulfamethoxazole prophylaxis.
Early follow-up	Sinonasal symptoms, epistaxis frequency, fatigue, cutaneous lesions, and neuropathic symptoms improved or stabilized; renal function remained clinically stable.

Laboratory evaluation revealed elevated inflammatory markers and a positive c-ANCA with PR3 specificity (Table 2). Urinalysis demonstrated microscopic hematuria and proteinuria, raising concern for renal involvement.

**Table 2:** Laboratory evaluation Pertinent laboratory results above revealing a positive c-ANCA, elevated inflammatory markers, anemia secondary to chronic inflammation, and a urinalysis showing proteinuria with microscopic hematuria.

Test	Result	Normal range
c-ANCA (PR3-ANCA)	Positive (high titer)	Negative
p-ANCA (MPO-ANCA)	Negative	Negative
Erythrocyte sedimentation rate	72 mm/h	0-30 mm/h
C-reactive protein	48 mg/L	<10 mg/L
Hemoglobin	10.2 g/dL	12-16 g/dL
WBC count	9,800 cells/µL	4,000-11,000 cells/µL
Creatinine	1.4 mg/dL	0.6-1.2 mg/dL
Urinalysis	Proteinuria, microscopic hematuria	Negative

**Figure 2:**
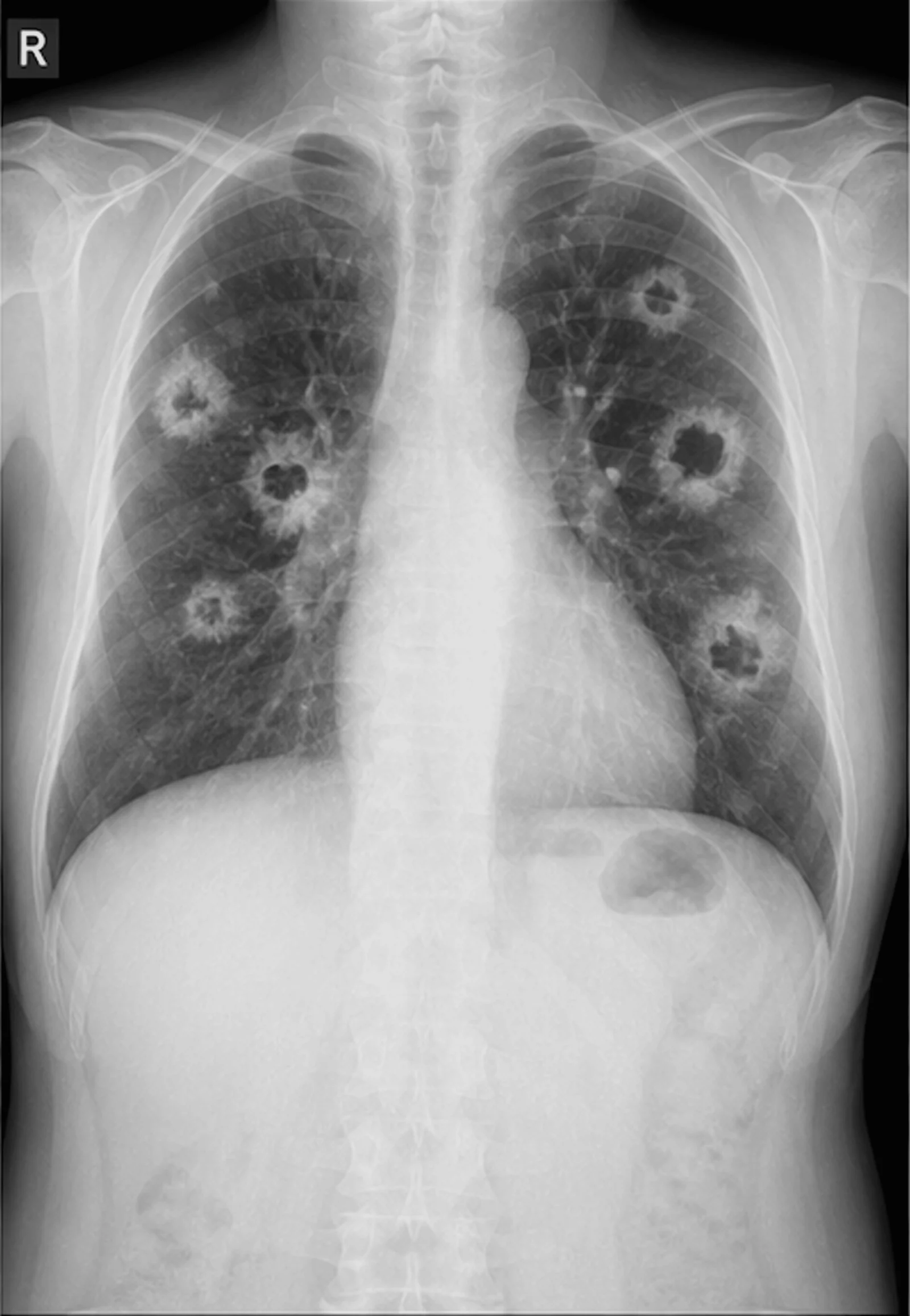
Chest X-ray Chest X-ray revealed multiple bilateral nodular opacities with cavitations.

**Figure 3:**
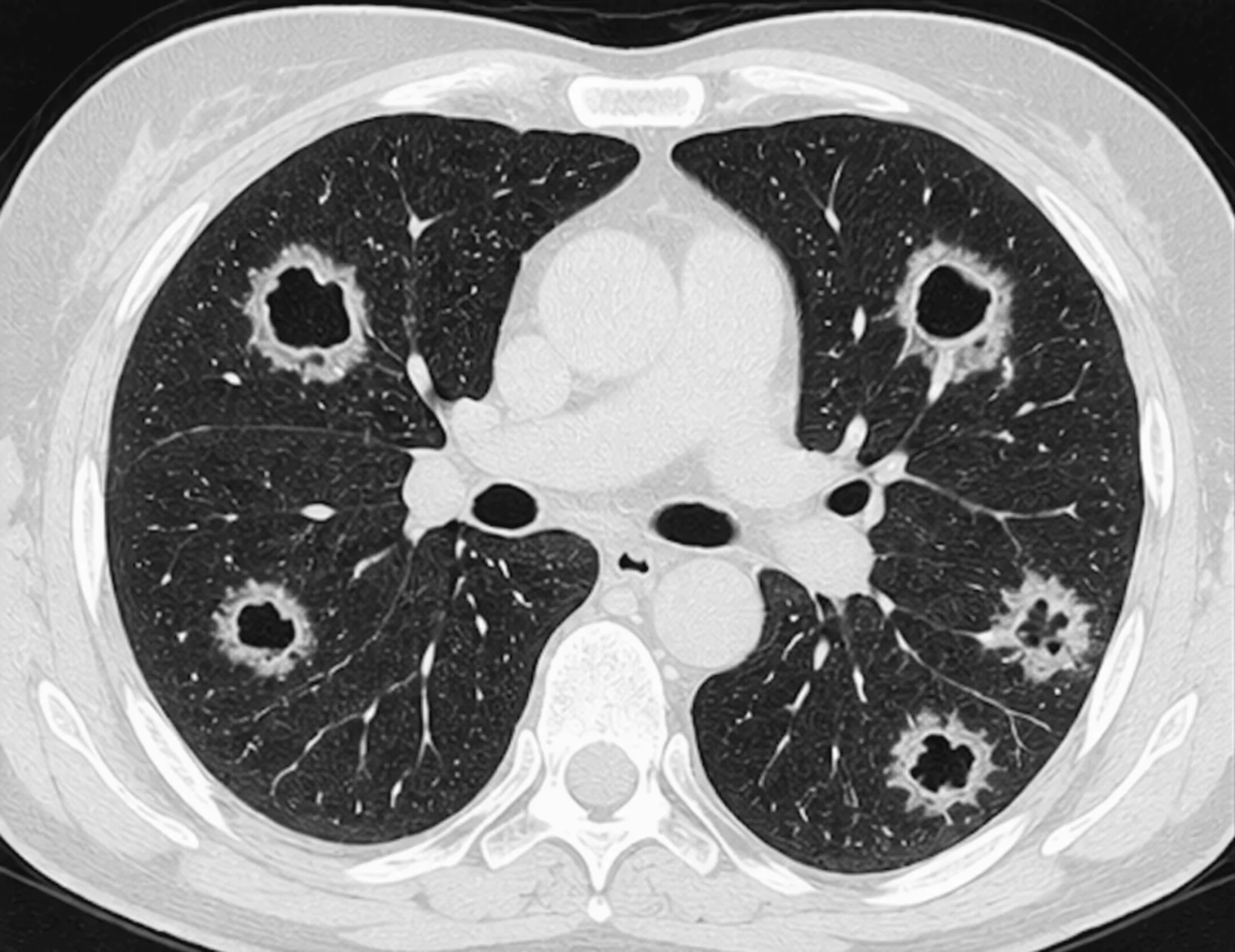
Computed tomography of the chest Computed tomography of the chest showed multiple bilateral cavitary pulmonary nodules of varying sizes with thick, irregular walls without evidence of mediastinal lymphadenopathy or pleural effusion.

**Figure 4:**
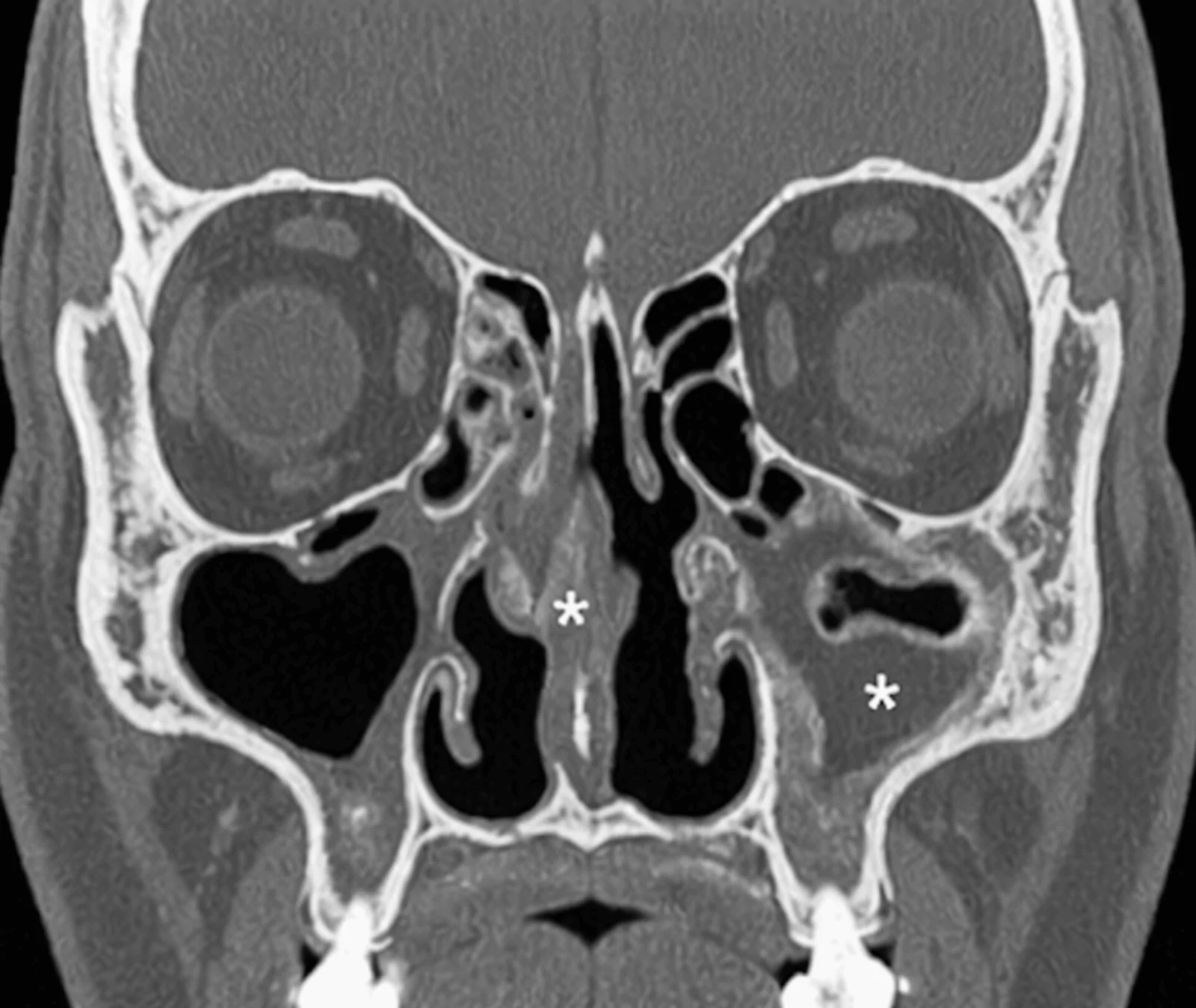
Computed tomography of paranasal sinuses Mucosal thickening and soft tissue in the nasal cavity and paranasal sinuses with bony destruction of the nasal septum (asterisk) and adjacent bony septations

Chest radiography showed multiple bilateral nodular opacities, some with cavitation (Figure 2). Non-contrast computed tomography (CT) of the chest demonstrated bilateral cavitary pulmonary nodules without mediastinal lymphadenopathy or pleural effusion (Figure 3). CT of the paranasal sinuses revealed mucosal thickening, soft tissue masses, and bony destruction of the nasal septum (Figure 4).

**Figure 5:**
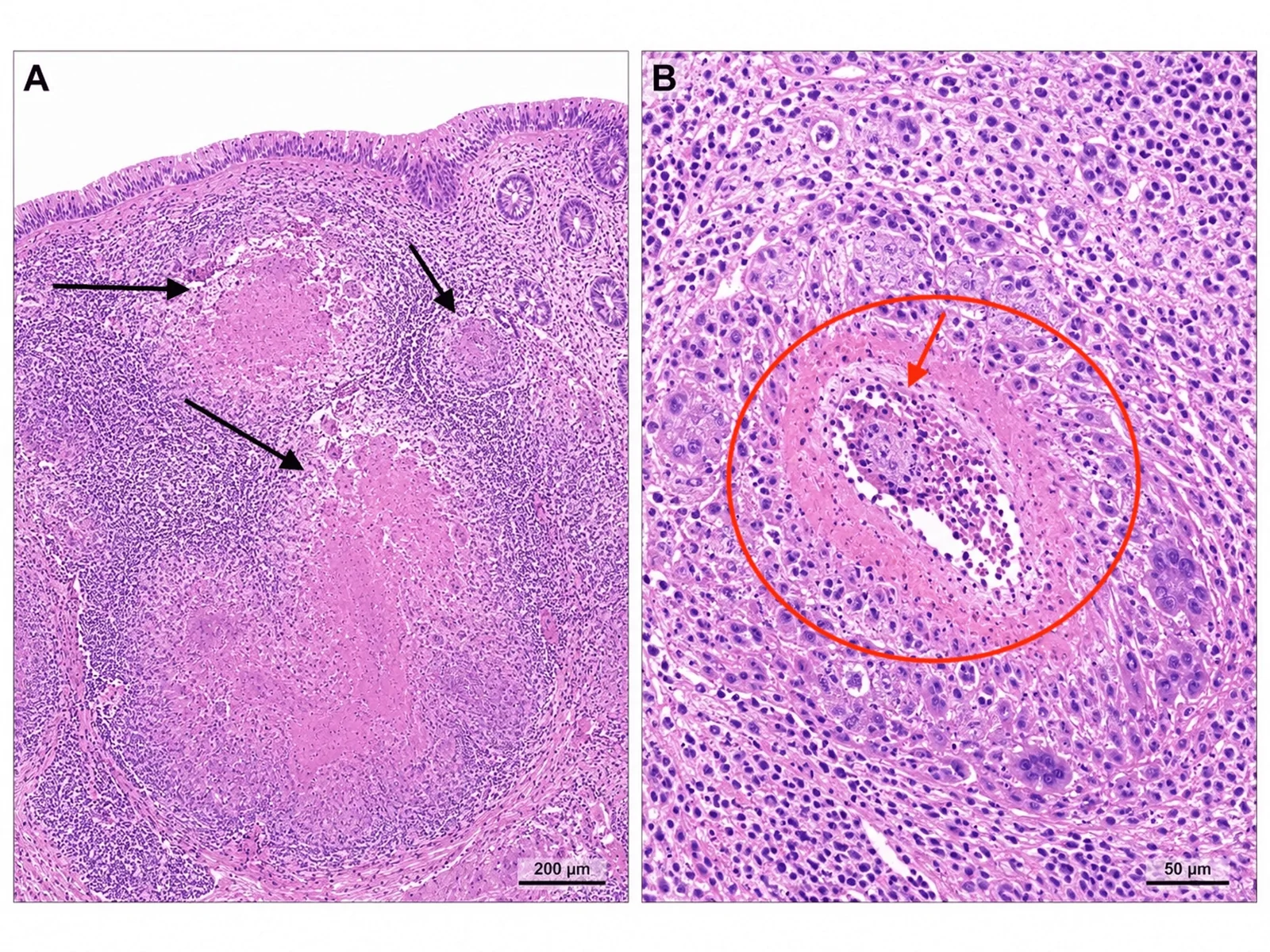
Histopathology The image on the left, labeled "A," is showing a low-power hematoxylin and eosin-stained image demonstrating sinonasal mucosa with necrotizing granulomatous inflammation and irregular necrosis (black arrows). The higher-power image on the right, labeled "B," is showing a small to medium vessel vasculitis with transmural inflammatory involvement (red arrow) and granulomatous inflammation (red circle); a histopathologic pattern supportive of granulomatosis with polyangiitis.

Histopathologic examination of a nasal biopsy demonstrated necrotizing granulomatous inflammation with vasculitis involving small- and medium-sized vessels, confirming the diagnosis of GPA (Figure 5). 

The differential diagnosis included refractory chronic rhinosinusitis, bacterial or fungal infection, tuberculosis, sinonasal malignancy, metastatic disease, sarcoidosis, IgG4-related disease, and other systemic vasculitides. GPA was favored because of destructive sinonasal disease, cavitary pulmonary nodules, palpable purpura, distal sensory symptoms, renal urinary abnormalities, PR3-ANCA/c-ANCA positivity, and confirmatory biopsy. Available histopathology did not report organisms or malignant cells. Bronchoscopy and lung biopsy were not documented, as the sinonasal biopsy provided diagnostic tissue in the clinical context (Table 3).

**Table 3:** Diagnostic reasoning and exclusion of competing diagnoses ANCA, antineutrophil cytoplasmic antibody; GPA, granulomatosis with polyangiitis.

Alternative diagnosis	Reason considered	Features arguing against/supporting GPA
Chronic allergic rhinitis or chronic rhinosinusitis	Long-standing congestion, epistaxis, and mucosal edema	Septal perforation, bony destruction, pulmonary cavitation, purpura, renal urinary abnormalities, and PR3-ANCA positivity exceeded uncomplicated rhinosinusitis.
Invasive fungal or mycobacterial infection	Cavitary lung nodules and destructive sinonasal disease can be infectious.	No fever, weight loss, or night sweats were reported; biopsy showed necrotizing granulomatous inflammation with vasculitis and did not report organisms. Dedicated microbiologic culture results were not available.
Sinonasal or pulmonary malignancy	Destructive local disease and pulmonary nodules may mimic cancer.	Multisystem vasculitic pattern, positive PR3-ANCA/c-ANCA, purpura, urinary abnormalities, and biopsy-confirmed vasculitis favored GPA.
Sarcoidosis	Granulomatous disease can affect lungs and upper airway.	Necrotizing granulomatous vasculitis, cavitary nodules, PR3-ANCA positivity, and destructive septal disease favored GPA.
IgG4-related disease	Can cause mass-like sinonasal disease	Cavitary pulmonary nodules, PR3-ANCA positivity, urinary findings, and necrotizing vasculitis were more consistent with GPA.
Microscopic polyangiitis or eosinophilic GPA	Other ANCA-associated vasculitides can involve the lungs, kidneys, skin, and nerves.	Granulomatous sinonasal destruction and PR3-ANCA/c-ANCA positivity supported GPA. No asthma/eosinophilic syndrome was reported.

The patient was referred to rheumatology and otolaryngology for multidisciplinary management. Induction therapy with high-dose systemic corticosteroids and rituximab was initiated. The exact rituximab dosing schedule was not available in the supplied record; if confirmed from the infusion record, it should be reported as either 375 mg/m^2^ weekly for four weeks or 1,000 mg intravenously on days 1 and 15, depending on the regimen used. Trimethoprim-sulfamethoxazole was prescribed for *Pneumocystis jirovecii* pneumonia prophylaxis, and close renal monitoring was arranged.

Following initiation of immunosuppressive therapy, the patient reported improvement in nasal obstruction, reduction in epistaxis frequency, and gradual improvement in fatigue and cutaneous purpuric lesions. Neuropathic symptoms stabilized without progression of weakness. Renal function remained stable on follow-up laboratory monitoring, and urinalysis was followed for resolution of hematuria and proteinuria. She continued outpatient rheumatology care for the completion of rituximab induction and steroid taper, otolaryngology follow-up for septal perforation and sinonasal surveillance, pulmonology follow-up with interval chest imaging to monitor cavitary nodules, and nephrology surveillance for possible evolving glomerulonephritis. At early follow-up, there was no reported hemoptysis, respiratory deterioration, or need for hospitalization, and the treatment plan remained focused on remission induction and prevention of renal and pulmonary progression. Long-term repeat chest CT findings, ANCA trend, quantitative urine protein response, steroid taper details, remission status, and treatment completion outcomes were not available at the time of manuscript preparation.

## Discussion

This case illustrates a common diagnostic pitfall rather than a previously undescribed clinical syndrome: GPA may smolder for years as refractory rhinosinusitis before systemic features reveal the underlying vasculitis. The diagnostic delay likely reflected anchoring to chronic rhinitis/rhinosinusitis in a patient whose early symptoms were common, non-specific, and initially only incompletely responsive to topical or allergy-directed therapy.

The differential diagnosis for chronic rhinosinusitis with systemic features is broad and includes allergic rhinitis, invasive fungal sinusitis, sinonasal malignancy, sarcoidosis, tuberculosis, IgG4-related disease, and other systemic vasculitides [1]. In this patient, the combination of destructive sinonasal disease, pulmonary cavitation, positive c-ANCA, and confirmatory histopathology narrowed the diagnosis to GPA.

Several clinical features should have prompted earlier reconsideration of the diagnosis. Recurrent epistaxis, nasal obstruction refractory to conventional therapy, septal perforation, palpable purpura, distal sensory symptoms, cavitary pulmonary nodules, and urinary abnormalities are not expected in uncomplicated chronic rhinosinusitis. When these findings coexist, the diagnostic frame should shift from localized sinonasal disease to systemic inflammatory, infectious, malignant, and vasculitic processes.

The pulmonary findings were particularly important. Cavitary pulmonary nodules are a recognized manifestation of GPA and may be mistaken for infection or malignancy [5]. The patient’s remote spontaneous pneumothorax was not definitively attributable to GPA; in this case, the prior pneumothorax is best interpreted as a historical clue that may be relevant but not diagnostic.

Renal and neurologic findings also required careful interpretation. Microscopic hematuria and proteinuria, with a creatinine of 1.4 mg/dL, raised concern for renal involvement, but a renal biopsy and quantitative proteinuria were not available. Similarly, distal sensory loss suggested peripheral neuropathy, but electromyography, nerve conduction studies, and formal neurologic consultation were not documented. These limitations were added to avoid overstating the degree of organ confirmation.

Cutaneous manifestations such as palpable purpura reflect small-vessel involvement and may precede renal disease [3]. The patient’s history of spontaneous pneumothorax likely reflects pulmonary parenchymal fragility related to cavitating nodules, a recognized though uncommon complication of GPA [5,6].

Untreated GPA carries a high mortality rate due to renal and pulmonary failure; however, modern immunosuppressive therapy has dramatically improved outcomes, with remission achieved in the majority of patients [7]. Rituximab is now a preferred induction agent, particularly in patients with relapsing disease or contraindications to cyclophosphamide [7].

The emphasis on this case lies in its prolonged presentation as refractory chronic rhinitis with dermatologic vasculitis and a prior pneumothorax, emphasizing how subtle ENT and skin findings may serve as early indicators of a potentially life-threatening systemic disease.

This case has several limitations. The exact duration between the first sinonasal symptoms, the prior nasal biopsy, the definitive biopsy, and treatment initiation could not be fully reconstructed from the supplied record. The prior nasal biopsy result, exact PR3-ANCA titer, urine protein quantification, complement levels, anti-nuclear antibody testing, renal biopsy, skin biopsy, EMG/NCS, bronchoscopy, repeat chest imaging, ANCA trend, and long-term remission status were not available. Clinical photographs of purpura were not obtained. 

## Conclusions

GPA should be considered in patients with persistent or treatment-refractory sinonasal symptoms when epistaxis, septal perforation, pulmonary cavitation, palpable purpura, neuropathic symptoms, or urinary abnormalities are present. This case demonstrates how a condition initially resembling chronic rhinosinusitis may represent systemic necrotizing vasculitis requiring urgent multidisciplinary evaluation.

The patient’s diagnosis was supported by PR3-ANCA/c-ANCA positivity, destructive sinonasal and pulmonary imaging, urinary abnormalities, and biopsy-proven necrotizing granulomatous vasculitis. Although this single case cannot establish long-term outcome benefit, existing evidence supports early recognition and prompt immunosuppressive therapy to reduce the risk of irreversible renal and pulmonary injury.
